# Surveillance for respiratory infectious diseases caused by 6 common viruses in a recruit training site in the Northern region of China

**DOI:** 10.1186/s40779-017-0120-y

**Published:** 2017-04-01

**Authors:** Wei-Wei Chen, Wen Xu, Yang-Xin Xie, Yun-Hui Zhang, Dan Wu, Fu-Sheng Wang, Min Zhao

**Affiliations:** 1 Treatment and Research Center for Infectious Diseases, 302 Hospital of PLA, Beijing, 100039 China; 2 Center for Medical information, 302 Hospital of PLA, Beijing, 100039 China

**Keywords:** Recruit, Surveillance, Respiratory infectious diseases

## Abstract

**Background:**

Recruit training sites are places with a high incidence of respiratory infectious diseases. Effective surveillance for acute respiratory infectious diseases in a recruit training site is an important way to prevent disease outbreaks.

**Methods:**

Eight hundred recruits (722 males and 78 females) enlisted in autumn 2015 received a background survey within 24 h of settlement at the recruit training site, including their general personal information, vaccination history, mental status and clinical symptoms. Then, nasopharyngeal swabs of these recruits were collected to detect common respiratory pathogens [influenza virus type A, influenza virus type B, adenovirus (Adv), human respiratory syncytial virus, human bocavirus and human metapneumovirus] by PCR. In addition, fasting venous blood was collected in the morning for adenovirus IgG antibody detection. During the three months of training, the recruits were monitored for symptoms of respiratory infection, and nasopharyngeal swabs were collected from those with an axillary temperature ≥38 °C and other respiratory symptoms within 4 h of symptom onset. Samples were further examined by PCR.

**Results:**

Among the 795 effective nasopharyngeal swab samples collected during survey, two cases of group C type 1 adenovirus were identified by PCR. During the 3 months of training, fever and respiratory symptoms occurred in 39 recruits (incidence rate of 4.9%) and 5 cases of adenovirus were detected (positive rate of 12.8%). Genotyping showed 3 cases of type 4 adenovirus and 2 of type 3 adenovirus. No type 7, 14 or 55 adenovirus was detected. The Adv-IgG positive rate of recruits was 48.2%. Among the 5 Adv positive cases with fever and respiratory symptoms, 4 were Adv-IgG positive.

**Conclusion:**

The pathogen carrier rate in recruits was low, and only group C adenovirus, which causes mild infection in humans, was detected. No respiratory outbreak was observed at the recruit training site, and sporadic cases were mainly caused by type 3 and type 4 adenoviruses.

## Background

Due to their high incidence, ease of spread and the difficult nature of prevention and treatment, acute respiratory diseases (ARDs) are major threats to armies worldwide [1]. Therefore, ARDs have become an important part of military medical study. Recruit training is a necessary step and effective way for improving the quality of troops and guaranteeing their combat capacity. During the training period, intensive physical training and changes in lifestyle, living environment and social support expose the recruits to different types of injuries, including physical injuries and ARDs [2, 3]. Furthermore, due to dramatic weather changes, the incidence of ARDs is higher in autumn and winter when recruitment in China usually occurs, and recruits are sensitive to respiratory infections. In recent years, a number of massive ARDs epidemics associated with adenovirus infection have occurred at recruit training sites [4–8], causing significant damage to soldiers’ health and imposing a huge burden of health protection and disease prevention in the troops [9].

Monitoring respiratory symptoms and the causal pathogens is an effective way to understand infectious pathogens, monitor the epidemic dynamics of infectious diseases, and prevent the occurrence of respiratory tract infectious diseases during the recruit training period. From the early 1970s to the late 1990s, the United States (US) military had implemented the ARDs surveillance program [10]. This program helped track and monitor all hospitalized recruits with ARDs. Over the past decade (2000–2014), the US military insisted on this program and has monitored patients with uncomplicated febrile ARDs (such as non-hospitalized patients). This program has been implemented for nearly 50 years, and it has been immensely valuable in identifying clusters of respiratory diseases among U.S. Army trainees [10]. Surveillance of febrile respiratory diseases was introduced in 2013 in China, when the ARDs pathogens surveillance network laboratories and surveillance sites were established [9].

To prevent respiratory infection outbreaks and investigate the causal pathogens for infections that occur in recruits, we monitored respiratory symptoms and performed respiratory infection surveillance in 800 recruits enlisted in autumn 2015 at a Chinese recruit training site.

## Methods

### Study design and subject selection

This study was an assigned mission. All 800 recruits at a recruit training site in the Hebei province of China enlisted in autumn 2015 were enrolled in this study. The background survey was conducted within 24 h of settlement at the training site in a cross-sectional manner. The period of ARDs surveillance was from September to December 2015 (the same as the training duration). An individual was considered a pathogen carrier if the nasopharyngeal swabs were positive for at least one pathogen without overt symptoms.

### Collection of background information of recruits

A questionnaire was prepared in a unified form to collect background information from the recruits within 3 days after arrival at the training site (the questionnaire included birth place; place of recruitment; history of infectious diseases; vaccination history; nutrition condition; mental state; and whether suffering from fever, cough, runny nose, sore throat or other respiratory symptoms within 3 days). The questionnaire survey was conducted in the dormitory of the recruits. The interviewers gave every recruit the questionnaire and explained every term that the recruits did not understand. The duration of this questionnaire survey was not limited allowing everyone sufficient time to fill out the form. All recruits could freely ask questions of the interviewers, but discussion among the recruits was not allowed. The interviewers were well trained health technicians.

### Collection of blood samples and swabs

Within 3 days of recruit settlement at the training site, 10 ml of fasting venous blood was collected in the morning by professional nurses, and serum was separated and stored at −20 °C to detect adenovirus IgG. Nasopharyngeal swabs were collected following appropriate procedures, and they were placed in universal transport medium. These samples were sent to the laboratory immediately for testing or storage at −80 °C.

### Adenovirus IgG detection

The adenovirus IgG in serum was detected using semi-quantitative ELISA kits (catalog number: 20160301) provided by Beijing Beier Biological Engineering Co. Ltd. (Beijing, China), according to the manufacturer’s instructions. The cut-off absorption value for this kit was defined as the mean value of negative controls plus 0.1. A positive sample was defined as having an absorption value higher than the cut-off value.

### Surveillance of febrile respiratory symptoms

During the training period, the body temperature of the recruits was monitored, and individuals with a temperature ≥38 °C and other respiratory symptoms (cough, sputum, nasal congestion, runny nose, sore throat, headache, and fatigue) were isolated at a separate ward. Nasal swabs of these recruits were collected within 4 h of symptoms onset. Case report forms (including body temperature, cough, runny nose, sore throat and other symptoms, as well as the respiratory rate, pulse, blood pressure, etc.) were completed and sent to the central laboratory of the Institute of Disease Control and Prevention, Academy of Military Medical Sciences (Beijing, China). Nasal swabs were sub-packaged promptly and stored in a refrigerator at −80 °C; they were then sent to the network laboratory at 302 Hospital of PLA (Beijing, China) for further testing.

### Pathogen detection

The various pathogens were detected by real-time quantitative PCR from the collected nasopharyngeal swabs. Pathogenic genomes were extracted from the nasopharyngeal swabs using a Viral Genomic DNA/RNA Extraction Kit (Catalog No. DP315, Tiangen Biotech, Beijing, China), and they were assessed by Influenza A/B Viral RNA Detection Kit (Catalog No. P0101C-24, MOKOGENE, Nanjing, China) and Respiratory Syncytial Virus (RSV)/Adenovirus/Human Metapneumovirus/Human Bocavirus Nucleic Acid Detection Kit (Catalog No. P0304-24, MOKOGENE, Nanjing, China). Adenovirus positive samples were re-tested using the One Step PrimeScript RT-PCR Kit (Catalog No. RR064A, Takara, Shiga, Japan) with common adenovirus primers to confirm the results. Samples positive for influenza virus A, influenza virus B or adenovirus were further tested with the genotyping primers shown in Table 1. All reactions were performed on an ABI 7500 PCR System (Applied Biosystems, Foster City, CA, USA). Two segments of the hexon gene of HAdV were amplified by PCR using 2 pairs of specific primers (Table 2), and the resulting products were sequenced by Beijing AuGCT Biotechnology Co., Ltd. (Beijing, China). Then, a sequence homology search was performed to identify the subgroup of adenovirus *via* the BLAST program at NCBI (http://blast.ncbi.nlm.nih.gov/Blast.cgi, 2015).

**Table 1:** Primers and probes used for pathogen detection in acute respiratory infections

Pathogen	Name of primer	Sequence (5′-3′)
Influenza A virus	QFluA-F	GAC CRA TCC TGT CAC CTC TGA C
	QFluA-R	GGG CAT TYT GGA CAA AKC GTC TAC G
	QFLuA-Probe	FAM- TGC AGT CCT CGC TCA CTG GGC ACG –BHQ1
Influenza B virus	QFluB-F	TCC TCA ACT CAC TCT TCG AGC G
	QFluB-R	CGG TGC TCT TGA CCA AAT TGG
	QFLuB-Probe	FAM- CCA ATT CGA GCA GCT GAA ACT GCG GTG -BHQ1
Influenza A seasonal H1	QH1F	AAC ATG TTA CCC AGG GCA TTT CGC
	QH1R	GTG GTT GGG CCA TGA GCT TTC TTT
	QH1-Probe	FAM-GAG GAA CTG AGG GAG CAA TTG AGT TCA G-BHQ1
Influenza A seasonal H3	QH3F	ACC CTC AGT GTG ATG GCT TCC AAA
	QH3R	TAA GGG AGG CAT AAT CCG GCA CAT
	QH3-Probe	FAM-ACG CAG CAA AGC CTA CAG CAA CTG T-BHQ1
Human adenovirus group B	qHAdV-UniF	TTTGAGGTYGAYCCCATGGA
	qHAdV-UniR	AGAASGGTGTRCGCAGGTA
	qHAdV-UniProbe	FAM-ACCACGTCGAARACTTCGAA-BHQ1
Human adenovirus type 3	qHAdV3-F	GGGAGACAATATTACTAAAGAAGGTTTGC
	qHAdV3-R	CAACTTGAGGCTCTGGCTGATA
	qHAdV3-Probe	FAM-CACTAC“T*”GAAGGAGAAGAAAAGCCCATTTATGCC
Human adenovirus type 4	qHAdV4-F	AAGCCAACCTGTGGAGKAACT
	qHAdV4-R	TGTTGGCCGGTGTGTATTTG
	qHAdV4-Probe	FAM-CTSTATGCCAATGTTGCCCTCTATTTGCCT-BHQ1
Human adenovirus type 7	qHAdV7-F	GAGGAGCCAGATATTGATATGGAATT
	qHAdV7-R	AATTGACATTTTCCGTGTAAAGCA
	qHAdV7-Probe	FAM-AAGCTGCTGACGCTTTTTCGCCTGA-BHQ1
Human adenovirus type 11/55	qHAdV55-F	CGGAGCAGCCAAATCAGAA
	qHAdV55-R	CATGAGTGTCTGGAGTTTCCAAAT
	qHAdV55-Probe	FAM-TGCGGCATCACAGAAAACAAACTTAAGTC-BHQ1
Human adenovirus type 14	qHAdV14-F	GAAAATCATGGTGTGGAAGATGAA
	qHAdV14-R	CAAGCTTGGTCTCCATTTAACTGA
	qHAdV14-Probe	FAM-ACGGCATCGGTCCGCGAACA-BHQ1
Internal reference	QRnaseP-F	AGA TTT GGA CCT GCG AGC G
	QRnaseP-R	GAGCGGCTGTCTCCACAAGT
	QRnaseP-P	FAM-TTCTGACCTGAAGGCTCTGCGCG-BHQ1

**Table 2:** Primers for the amplification of a finger-print sequence of human adenovirus

Name of primer	Sequence (5′-3′)	Product length (site in the hexon gene of HAdV)
HAdVT1-F HAdVT1-R	CCATAYTCBGGYACDGCYTAC TGTARTTGGGTCTGTTDGGCAT	595 bp (474–1068)
HAdVT2-F HAdVT2-R	GCDGTAGACAGYTATGATCC GTGRAARGGCACRTARCGDCC	498 bp (1257–1754)

### Data analysis

All data were entered into a database, and logical errors were detected and corrected using the Excel program (Microsoft Office EXCEL 2003, WA, USA). The corresponding frequency, constituent ratio, or incidence was calculated. Measurement data consistent with a normal distribution (tested by the Kolmogorov-Smirnov test) and expressed as $\overset{-}{x}$± *s* (calculated by the Execl program).

## Results

### Background information of recruits

Background information was collected from the 800 recruits. There were 722 males and 78 females, who were aged 18.9 ± 1.69 years. Most recruits came from Hebei (199, 24.9%), Shandong (102, 12.8%), Jiangsu (196, 24.5%), Shanxi (99, 12.4%) and Hunan (204, 25.5%). The ratio of recruits from urban areas to those from rural areas was 1:1.12 (378:422). Basic information, lifestyle, and medical history of the assessed recruits are summarized in Table 3.

**Table 3:** Background information for the recruits

Item	Basic information
Male/Female (*n*)	722/78
Age(year)	18.9 ± 1.69
Life style [*n* (%)]	
Smoke	360(45)
Drunk	352(44)
Medical history [*n* (%)]	
Tonsillectomy	14(1.8)
Recurrent respiratory infection	37(4.6)
History of measles	20(2.5)
Chicken pox	241(30.1)
Influenza vaccination^a^	9(1.1)
National planned vaccination rate	758(94.8)

^a^means received influenza vaccination in the last 2 years

### Pathogen identification

None of the recruits had fever or respiratory symptoms when nasopharyngeal swabs were collected for the background survey. A total of 795 samples of nasopharyngeal swabs were tested, and 2 cases were adenovirus-positive as assessed by real-time PCR. None of the samples was positive for influenza virus A, influenza virus B, RSV, human metapneumovirus or human bocavirus. After genotyping and sequencing, the 2 adenovirus-positive cases were identified as group C type 1 adenovirus. The labels of 5 samples were missing or could not be recognized clearly; these samples were not tested.

### Monitoring of cases with febrile respiratory symptoms

During the three months of training, 39 recruits had febrile respiratory infection symptoms (incidence rate of 4.9%) with an average temperature of 38.7 °C (38.0–40.2 °C). The main symptoms included cough (34, 87.2%), expectoration (15, 38.4%), sore throat (27, 74.3%), headache (27, 69.2%), fatigue (29, 74.3%), runny nose (19, 48.0%), muscle pain (8, 20.0%) and diarrhea (3, 7.7%). No chest distress, chest pain or suffocation was observed. All cases had fever abatement within 3 days after supportive treatment. The disease course was 2–7 days.

### Pathogen detection in cases with febrile respiratory symptoms

Five of 39 nasopharyngeal swabs were adenovirus-positive (positive rate of 12.8%). Further adenovirus genotyping showed that there were 3 cases with group B adenovirus type 4 and 2 with adenovirus type 3. No type 7, 14 or 55 adenovirus was detected. Influenza virus was not detected in any cases with febrile respiratory symptoms.

### Investigation of serum adenovirus immunoglobulin G (IgG) of recruits

Because of missing or blurred label or an inadequate sample volume, 70 samples were not tested. Adenovirus IgG (Adv-IgG) in 730 serum samples was tested, and 352 samples were Adv-IgG positive (positive rate of 48.2%). Further analysis of the Adv-IgG of 39 cases with febrile respiratory symptoms showed that among the 5 adenovirus-positive cases, 4 were Adv-IgG positive (positive rate of 80%). Among the 34 adenovirus-negative patients, 13 cases were Adv-IgG positive (positive rate of 38.2%, Table 4).

**Table 4:** Adenovirus IgG in serum samples and adenovirus in nasopharyngeal swabs from recruits with febrile respiratory infection symptoms [*n* (%)]

IgG	All recruits	Recruits with ARDs and AdV infection^a^	Recruits with ARDs without AdV infection^b^
IgG^+^	352 (48.2)	4 (80)	13 (38.2)
IgG^−^	378 (51.8)	1 (20)	21 (61.8)
Total	730 (100)	5 (100)	34 (100)

^a^means the recruits who acquired ARDs caused by AdV infection confirmed by a PCR test

^b^means recruits who acquired ARDs without AdV infection confirmed by a PCR test

## Discussion

We performed a general survey on pathogens responsible for respiratory infectious diseases at a recruit training site for the first time. To improve the data quality, we selected commercial kits for preliminary screening. The results showed that the pathogen carrier rate in recruits without respiratory symptoms was very low, and among 795 nasopharyngeal swab samples, only two were adenovirus-positive; they were identified as group C type 1 adenovirus with low virulence.

During the 3 months of training, 5 cases of adenovirus were detected among 39 recruits with fever and respiratory symptoms, indicating that adenovirus is the major causal pathogen for respiratory infectious disease at recruit training sites. According to retrospective studies on respiratory infections in troops during the past 25 years in the USA, the most common ARDs pathogens in troops are adenovirus, influenza (parainfluenza) viruses, respiratory syncytial virus, metapneumovirus, rhinovirus, human coronavirus, mycoplasma pneumonia and Chlamydia pneumonia [10]. As early as the World War I, “Spanish flu” occurred in U.S. troops (1917–1918), and studies exploring the prevention and control of ARDs were initiated and have continued i.e., for approximately 100 years. In China, such surveillance only started 3 years ago. There is still a lack of comprehensive information regarding the spectrum of febrile respiratory diseases. During the Vietnam War period (1965–1970), RSV infection ranked second among the febrile respiratory diseases affecting US troops, surpassed only by adenovirus infection (21.0%) [11]. According to a survey [12], RSV accounts for 0.73% of pathogens of upper respiratory tract infections in Chinese troops, less than the infection rates of mycoplasma pneumonia and Legionella. However, we did not detect RSV positive cases in this investigation, nor did we find metapneumovirus or bocavirus. These viruses are common in upper respiratory tract infections that affected children, but they are less common in Chinese adults [13, 14], which suggests that these pathogens will not be detected in future background investigations and pathogen surveillances.

Adenovirus infection usually spreads quickly, which may cause seasonal epidemics. In foreign military camps, most febrile respiratory diseases are caused by human adenovirus types 4, 7, 14 and 55 infections [10, 15]. In Chinese troops, most pathogens that have caused massive epidemics in recent years have been adenovirus types 7, 14 and 55 [16]; of these, type 55 accounted for more than 50% of all infections. In the present study, human adenovirus type 55 was not detected, and massive respiratory infection outbreaks did not occur. According to several adenovirus infection outbreaks in the past, diseases caused by adenovirus type 7 are mild and, compared with infection by adenovirus type 55, their incidence of pneumonia is low. In the event of pneumonia, it is mainly mild, and has a short course and good prognosis, without severe cases [4–7]. In this study, group B adenovirus types 4 and 3 are the main types causing febrile respiratory diseases. According to previous reports, adenovirus types 4 and 7 are the main types that caused mass outbreaks of upper respiratory tract diseases in US troops in the 1970s, while adenovirus type 4 infections caused the deaths of many soldiers [10, 17]. Usually, symptoms of HAdv-3 infection are mild, and we found upper respiratory infections in only two cases, without pneumonia or other complications.

The recruits in the training site were from the central and northern parts of China, and an adenovirus IgG positive rate of 48.2% was obtained. Statistical data showed no difference in the IgG-positive rates between provinces, or between urban and rural areas (data not shown). The previously reported infection rate of adenovirus in the population is close to 50% [18, 19], but after detecting Adv-IgG in people with febrile respiratory symptoms, we found that among 4 of 5 Adv positive cases were Adv-IgG positive. Of note, more than 50 serotypes of human adenovirus can cause clinical symptoms, and there is no cross-protection between them [16]. We detected total IgG against adenovirus, and only type-specific neutralizing antibodies have a protective effect [19]. Therefore, although the Adv-IgG positive rate in the recruits was as high as 48.2%, mass infections of adenovirus types 4, 7, 14 and 55 may still occur, which can be explained by the multiple mass adenovirus epidemics that occurred in recent years. Specially, HAdV-55, derived from a recombination of adenovirus types 11 and 14, has caused a number of outbreaks in the troops in recent years [4–9]. According to the HAdV-55 infection epidemic situations in late 2011 and early 2012, over 6300 people were exposed to the virus, and nearly one thousand soldiers were infected, all of whom were young adults. There was severe illness in more than 30% of cases and one death [20]. However, no outbreaks have been reported in crowded nurseries and non-military institutions.

The USA is the sole country where recruits receive adenovirus vaccination. During Adv-4 and Adv-7 vaccination in the period from mid-1970s to 1999, the incidence of febrile respiratory diseases in US troops was greatly reduced; however, Wyeth Pharmaceutical stopped the production of adenovirus vaccines in 1996. Additionally from 1999 (when vaccines in stock were used up) to 2011, the incidence of febrile respiratory diseases rose to the level prior to vaccination. In the two years from 2011 to 2013, when adenovirus vaccine inoculation recovered, the burden of adenovirus infection diseases in American recruits was reduced 100-fold; These data strongly supported the production and administration of Adv-4 and Adv-7 vaccines to control febrile respiratory diseases, and suggested the implementation of continuous surveillance for emerging adenovirus subtypes [21]. To prevent and control massive adenovirus epidemics, the development and administration of vaccines should be included in the plans of the Chinese army to ensure that the intensive research should be done on the basic research and industrial production of vaccine, and the health economic value of vaccine should be evaluated at the same time.

Symptom and pathogen surveillance is the first step in ARDs prevention and control. Epidemiological surveys should be performed when the ARDs incidence rate is higher than the level in the same period from a year prior. The situation should be reported to the army and government leaders, and disease surveillance should be performed as early as possible to propose prevention and control strategies. This background information and pathogen surveillance mainly focused on flu and adenovirus infection that easily cause massive epidemics of respiratory infectious disease. No influenza virus A or B was detected, which is probably because community influenza virus did not spread to the military camp or maybe because of false negative results. Because the pathogen detection rate in this study was low (12.8%), no pathogen was detected in 34 cases, which is likely due to the inappropriate time and method of collecting nasopharyngeal swabs in addition to the sensitivity of detection reagents. Five cases of group B adenovirus detected in patients with febrile respiratory diseases in this study were not associated with the 2 cases of group C adenovirus type 1 found in the background survey. Epidemiological analysis revealed that the five patients were sporadic cases who had a history of contact with outsiders. From the end of 2011 to early 2012, Adv 55-type mass outbreaks occurred twice. The veterans were infected outside, and the recruits and veterans had cross spreading. In addition, due to off-site training, spreading across camps occurred [20]. Epidemiological survey and whole-genome sequencing revealed that the two massive outbreaks of adenovirus infections in different places during the period from December 2012 to February 2013 were caused by the same strain of adenovirus type 7 [22]. Recruits infected in the first outbreak were in close contact with an individual from the training site where the second outbreak occurred during their hospitalization, and the latter brought the virus back to the other recruit training camp, causing another Adv-7 epidemic in a distant training site [22]. Therefore, detailed epidemiological survey and whole-genome sequencing can provide useful information for infection control and intervention. In this regard, we can learn from American troops. The respiratory surveillance program in the US includes all cases and disease courses, and it especially implements self-isolation management of patients who temporarily stop training due to febrile respiratory diseases. For example, patients are isolated for at least 8 h in a special separated room or outpatient clinic, or their scope of activities is limited upon their returned to training.

The population in this study was from only 1 recruit camp in Hebei province, and the recruits came from central and northern China. Therefore, the results might not be generalizable to recruit camps in other locations. More studies are warranted to characterize the pathogen distribution profile among recruit camps and provide more information for ARDs control in recruits.

## Conclusions

The pathogen carrier rate in the assessed recruits was low, and only group C adenovirus, which causes mild infection in humans, was detected. No respiratory outbreak was observed in the recruit training site, and sporadic cases were mainly caused by type 3 and type 4 adenoviruses. The Adv-IgG positive rate in recruits was almost 50%. Although this is only a pilot study, it provides a reference for future surveillance that covers all recruit training sites of the Chinese Army, and it is of great significance in preventing the spread of massive infectious disease outbreaks among recruits.

## Abbreviations

Adv: Adenovirus
ARDs: Acute respiratory diseases
HAdv: Human adenovirus
ILI: Influenza like illness
RSV: Respiratory syncytial virus
